# Can the nutritional geometric framework unveil how macronutrients in pollen shape solitary bee foraging and survival?

**DOI:** 10.1098/rsos.251093

**Published:** 2025-08-13

**Authors:** Jaya Sravanthi Mokkapati, Natalie Boyle, Pierre Ouvrard, Adrien Sicard, Christina M. Grozinger

**Affiliations:** ^1^ Department of Entomology, Center for Pollinator Research, Huck Institutes of the Life Sciences, Pennsylvania State University, University Park, PA 16802, USA; ^2^ Uppsala Biocenter, Department of Plant Biology, Uppsala Box 7080, 750 07, Sweden

**Keywords:** pollinators, nutritional ecology, solitary bees, foraging behaviour, nutritional geometry

## Abstract

The nutritional geometric framework (NGF) hypothesizes that animals forage to achieve specific macronutrient ratios. Pollen, the primary source of protein and lipids for bees, varies in nutritional content across plant species. Studies suggest some bumblebee species forage based on species-specific macronutrient preferences, regardless of floral traits. Here, we examine whether females of the solitary bee species, *Megachile rotundata*, also follow the NGF when foraging. In a semi-field experiment using *Capsella* recombinant inbred plant lines with varying pollen protein-to-lipid (P : L) ratios, *M. rotundata* visits were positively associated with pollen protein content. In a laboratory-based study using synthetic diets with varying P : L ratios, female *M. rotundata* consumed the most and survived longest on diets with intermediate P : L ratios (5 : 1 and 10 : 1). Paired-choice experiments further revealed that bees regulated nutrient intake, converging on an average intake of 6.6 : 1 ± 1.6 P : L. These results suggest that *M. rotundata* exhibits a clear macronutrient preference and regulation behaviour, optimizing nutrient intake consistent with a functional nutritional target. Understanding how macronutrients influence foraging preferences and health outcomes in diverse bee species is crucial for understanding how plant-pollinator networks evolved and creating habitats beneficial for diverse pollinators.

## 1. Introduction

The nutritional geometric framework (NGF) predicts that animals will forage among possible dietary resources (e.g. flowering plants) to obtain a preferred nutritional ‘intake target’ which balances ratios of key macronutrients, such as carbohydrates, protein or lipids [1]. Most insect herbivores regulate their nutrient intake through behavioural and physiological adaptations [2]. Recently, the NGF has been applied to pollinators, such as bees, to determine if macronutrient ratios of floral resources, rather than floral traits such as colour, scent and size, drive foraging preferences [3]. While there is evidence from laboratory, semi-field and field studies that some bee species (e.g. *Bombus impatiens* bumblebees) preferentially forage to collect pollen with a specific protein-to-lipid (P : L) ratio [3–6], there are more than 20 000 bee species in the world [7], and it remains to be determined if and how other bee species regulate their nutritional intake. Furthermore, the relationship between nutritional foraging preferences and bee health, reproduction and survival outcomes remains unclear.

Pollen is the primary source of protein and lipids for bees and is essential for larval development and adult physiological functions such as vitellogenesis [1,8–11]. Most bee species collect pollen produced by flowering plants [12–14], yet concentrations of macronutrients vary significantly among plant species, typically ranging from 2 to 60% protein and 2 to 20% lipid [9]. This variability in nutrient composition across plant species should result in selection for bees to develop behavioural strategies to selectively forage across diverse plant species to fulfil their nutritional needs [15]. Failure to meet specific nutritional targets can compromise bees’ stress resilience, reproduction and overall fitness [16–18] and lead to increased mortality [19,20], ultimately impacting population dynamics. Indeed, the reduction of flowering plant abundance and diversity is thought to be a major contributor of declining populations of bee species across the world [21].

In previous studies, Vaudo *et al.* [4,6,11,22], using liquid diets, isolated pollen diets and flowering plant species, demonstrated that *Bombus impatiens* bumblebees selectively modulate their foraging behaviour to obtain pollen diets with a preferred P : L ratio. In the field, bumblebees collected pollen from multiple plant species to obtain a P : L ratio of 4 : 1, and their ability to collect large amounts of this pollen was correlated with colony size and production of reproductives [15]. Solitary bees, unlike eusocial species such as honeybees or bumblebees, do not support a colony or queen. Their foraging efforts are focused solely on egg-laying and provisioning brood, meaning each female must individually manage nutrition for her brood with limited flexibility in adjusting the P : L ratio. However, the degree to which different solitary bee species regulate their macronutrient intake under different conditions is largely unknown. In our recent study, we evaluated the foraging preferences of females from two solitary bee species, *Osmia cornifrons* (horned-faced mason bees) and *Megachile rotundata* (alfalfa leafcutting bees) using 20 *Capsella* recombinant inbred plant lines (inter-crossed populations of a pollinator-dependent *C. grandiflora* (*Cg*) and self-reproducing *C. rubella* (*Cr*) [23], in small indoor foraging arenas using clipped inflorescences with multiple flowers [24]. We found that only *M. rotundata* showed a significant preference for inflorescences from plants with higher pollen P : L content. We further confirmed in a laboratory-based choice assay between artificial diets of 10 : 1 and 1 : 1 P : L, that *M. rotundata* preferentially selected flowers offering the 10 : 1 P : L diet. This preference underscores the importance of nutritional quality in influencing *M. rotundata* foraging decisions. However, it is unclear whether nutrition plays a crucial role in *M. rotundata* foraging behaviour in natural habitats when resources are presented as whole plants, where vegetative and floral traits may influence attraction and visitation patterns. Furthermore, it remains to be determined if *M. rotundata* selectively collect pollen to achieve a specific macronutrient ratio (as is predicted by the NGF) or is simply foraging to maximize protein intake. Evaluating how macronutrient quantities and ratios influence foraging of different bee species in different environmental conditions is essential for understanding how plant-pollinator networks evolved and how to design and manage habitats that can best support specific bee species or pollinator communities.

In this study, we examined how the content of protein and lipid in food influence foraging preferences, feeding behaviour and survival in female alfalfa leafcutting bees, *M. rotundata*. First, in a semi-field experiment, using 16 recombinant inbred lines (RILs) of *Capsella* plants that varied in pollen protein and lipid concentrations, we assessed the foraging preferences of *M. rotundata* females for different floral traits, i.e. floral resource quantity (the number and size of flowers) and/or floral nutritional quality (pollen protein concentration, lipid concentration and P : L ratios) and the relationship between them. Second, we used NGF methodology to test and measure regulation of protein and lipid intake, using synthetic liquid diets differing in P : L ratios between 1 : 10 to 25 : 1 P : L, and measured the food consumption and survival of individual bees. Next, based on the results, we offered single bees a combination of two selected unbalanced diets (consisting of different P : L ratios) to investigate whether they regulate their intake of these macronutrients to reach a specific intake target. We hypothesized that the bees would adjust their consumption to achieve a P : L ratio that maximized their survival.

## 2. Material and methods

All experiments were conducted at Pennsylvania State University, University Park, Pennsylvania, USA.

### 2.1. Rearing and maintenance of bees

Cocoons of the managed solitary bee species in the family Megachilidae, *Megachile rotundata* (known as alfalfa leafcutting bees, or ‘ALCB’) were purchased from a commercial supplier (Watts Solitary Bees, Bothell, WA, USA) and stored as loose cocoons in the dark at 4°C until use. In summer 2022 and 2023, to stimulate emergence, cocoons were placed in petri dishes (4 g per dish) and acclimatized to a gradual increase in temperature at 23 ± 1°C for 8 hours to 29 ± 1°C in incubators (Thermo Scientific, USA) with 65 ± 5% relative humidity (RH). Following standard practices, on the 7th day of incubation, a strip of dichlorvos (Vapona™) pesticide was introduced to incubator for parasite control in developing cocoons (3/4th strip per 28.3 m^3^ [or 1000 ft^3^] of incubator space per Baird and Bitner [25]) and was removed on the 13th day, after which thoroughly aerated the incubator with additional fans. Once males started to emerge, dishes were observed twice daily, and individual adults were moved and maintained in small cages (10 × 5 × 7 cm plastic box with mesh) with equal number of males and females per cage and maintained in an environmental chamber set at 23 ± 1°C, 65 ± 5% RH and 16 : 8 hours light : dark (L : D) regime. Each box included a feeder containing 40% w/w sucrose solution provided ad libitum and a crumpled tissue paper for bees to take refuge. Emerged females were maintained in the cages for at least 3 days prior to introduction to the outdoor foraging arenas.

For laboratory experiments, only emerged females (approximately 1 to 3 days old, presumably mated individuals) were collected and kept in corresponding individual treatment boxes. See §§2.4 and 2.5 below for details of treatments. *Megachile rotundata* is a solitary, mass-provisioning bee in which females collect and deposit all larval food before oviposition [26], with no further parental care. Females forage continuously throughout their nesting cycle—each pollen collection supports a single brood cell, followed by egg-laying and cell closure with leaf tissues. As a result, adult nutrient regulation is tightly linked to offspring provisioning across the entire reproductive period. This life history suggests that nutrient-driven foraging behaviour is shaped by selection to meet both maternal maintenance and larval nutritional needs. Accordingly, only presumably mated female bees were tested, as they are solely responsible for nest construction and provisioning, while male bees’ activity is limited to insemination [27].

### 2.2. Plant growth and maintenance

Several recombinant interspecific plant lines were generated by crossing *C. grandiflora* (*Cg*) and *C. rubella* (*Cr*) by Sicard laboratory at Swedish University of Agricultural Sciences, Sweden (as described in [23]). Sixteen RILs with a relatively high degree of variation in phenotypic traits and good rate of germination (over 50%) were selected for the study along with their parental plant lines, *Cg* and *Cr*. Seeds from each *Capsella* plant line were germinated in the environmental chamber (16 : 8 h L : D, 23 ± 1°C and 65 ± 5% RH) and later grown in the greenhouse under controlled conditions. Detailed procedures for plant germination and growth are described in the electronic supplementary material. We measured the macronutrient concentrations (protein and lipid) in pollen from our experimental plants following our standardized laboratory protocols and presented in Mokkapati *et al.* [24]. Details of selected plant lines and their average macronutrient concentrations are presented in electronic supplementary material, table S1. A total of 144 plants (8 plants per plant line) were used for the bee foraging experiment.

### 2.3. Bee foraging assay—outdoor trials using *Capsella* plants

Foraging preferences of alfalfa leafcutting bees were assessed in field cages (6 × 6 × 3 m), covered with a 70% shade cloth. The experiment was conducted in two cages from 24 June to 6 July 2022, and each cage was equipped with a nesting box and flowering plants as described below.

**Figure 1.**
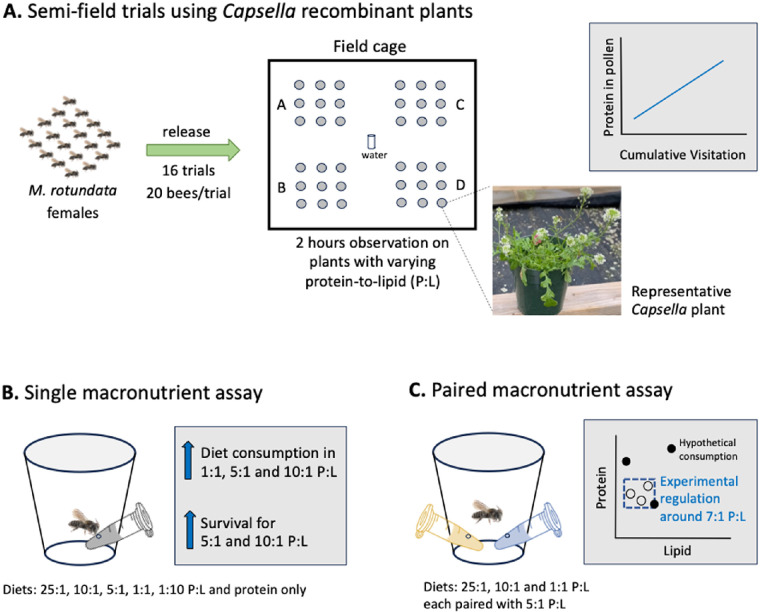
Schematic overview of the study using the solitary bee *Megachile rotundata*, including (A) foraging trials conducted in outdoor cages with *Capsella* recombinant plant lines, (B) laboratory-based macronutrient assay using single-diet treatments, and (C) paired-choice diet assay, to assess nutrient preferences.

On the day of the foraging assay, two plants each from 18 *Capsella* plant lines (16 RILs and 2 parents, *Cg* and *Cr*) were placed in four 3 × 3 grids (A–D). Figure 1 shows the schematic representation of the foraging assay in the field cage. Between trials, plant lines were randomly assigned to the grid locations.

For each foraging trial, 20 uniquely paint-marked mated *M. rotundata* females were released into the cage with the *Capsella* plants. Nest boxes were not provided in the cages, so bees were unable to provision nests during the experiment, which allowed us to isolate and observe foraging/feeding behaviour without the influence of nesting activity. The bees had been fed sucrose solution from artificial flowers while they were maintained in the cages in the laboratory. Bee visits were observed for 15 min at each grid (3 × 3 plants, A–D), rotating between grids for 2 hours (see figure 1A). Sixteen trials were completed, using a different cohort of 20 naive bees and two replicated plant arrays in each trial. A total of eight replicate plants per plant line were used for the whole experiment, with a minimum interval of 2 days between trials (involving the same plants) to allow new flowers to bloom providing fresh pollen or the plants to replenish their pollen.

When a bee touched the reproductive/flowering part of the inflorescence (excluding stem/plant pot) in the field cage, it was counted as a ‘visit’. Bee visits and time spent on the flowers of each plant line were continuously recorded for 2 hours using the MultiTimer application on a mobile phone. Additionally, bees were provided with sucrose and water in separate dishes at the centre of the cage, and bees were occasionally observed to drink from these dishes; thus, the bees were expected to forage on the plant for pollen but not for nectar (see electronic supplementary material for details on floral nectar traits). Bee foraging preferences were quantified by the number of bee visits to each plant and the time they spent on each inflorescence. While it is not entirely possible to differentiate pollen versus nectar visits, bees were observed to mostly forage for pollen (waggling their abdomen onto the flower, presumably to collect pollen on the scopa on the ventral side of their abdomens) and were not observed to probe the flower with their proboscis. The trial duration was standardized to 2 hours to observe enough visits while ensuring bees could explore flowers with intact pollen. During the trials, bees did not make any provisions inside the cage. Data analysis comparing the number of active bees per trial with the number of bees released into the cage yielded similar results, indicating that there were no density-dependent shifts in foraging activity among these bees.

During each trial, the number of inflorescences per plant was counted. Since there was a significant variation in the total number of inflorescences per plant line among trials (see electronic supplementary material, figure S1), we normalized our bee visitation data with the number of inflorescences present on each plant in each trial. Bee foraging preferences among the plant lines were compared directly by creating metrics at both the community and individual bee level, as in [22]. These metrics were cumulative visitation rates (CVR) which was defined by the total number of times a plant line was visited in 2 hours by all bees in the cages normalized for total number of inflorescences per plant line (visits/2 h/20 bees/total number of inflorescences) and individual visit duration (IVD) which was defined as the time spent by a single bee on each plant line (seconds/visit/bee) for pollen. While CVR reflects collective foraging activity that captures preference at group level, IVD provides insight into per-visit engagement or feeding effort. These metrics help to reduce the variation in the data caused by the number of bees in the environment and variation in flowering within plant lines.

Data on the nutritional values, i.e. pollen protein concentration, pollen lipid concentration and P : L ratio of the pollen was collected in a previous study [24] and thus represents average for these plant stocks. These plant stocks are RILs and were maintained under controlled conditions with minimal genetic drift to ensure consistency in plant genotypes within the set of the seeds collected. For the current study, we grew plants from the same seed stock for which the nutritional values in pollen were measured in [24]. Thus, the plants in the current study are expected to exhibit the same protein and lipid concentrations in pollen as previously measured.

### 2.4. Laboratory-based macronutrient assay—single diet

To characterize the preferred macronutrient ratios for *M. rotundata* female bees, a laboratory-based assay was performed using six artificial diets with altered P : L ratios: protein only, 25 : 1, 10 : 1, 5 : 1, 1 : 1 and 1 : 10 P : L, in 0.5 mol l^−1^ sucrose solution (figure 1B). Nutrient sources were sucrose (Sigma-Aldrich, St Louis, MO, USA) for carbohydrates, organic plant-based extract (Truvani, Nomolotus LLC, USA) for protein and 100% organic sunflower lecithin (Microingredients^®^, USA) for lipids (see electronic supplementary material for details). Sunflower lecithin was chosen as the lipid source due to its emulsifying properties, making it suitable for liquid diets. Since Truvani protein powder contains lipids at a 10 : 1 P : L, supplemental casein (Sigma-Aldrich, USA) was used to create the 25 : 1 P : L high protein diet, while pure casein was used for the ‘protein only’ reference diet. To prepare the diets, we mixed the lecithin with other components at corresponding P : L ratios, while stirring the mixture for approximately 1–2 hours under low heat to ensure a homogeneous solution. Liquid diets were used because the nutritional ratios can be easily controlled, and they are easy for the bees to access and ingest; such liquid diets have been previously used to assess protein, lipid and carbohydrate ratio preferences in honeybees and bumblebees [5,28].

A day before the experiment, newly emerged females were cold-anaesthetized and weighed to use their body mass as a covariate in data analyses and to control for effect sizes on diet consumption. Subsequently, bees were kept in individual treatment boxes (disposable plastic boxes with flat lids, 10 × 6 × 6 cm), one bee per box, and allowed to acclimatize to ambient conditions (16 : 8 h L : D, 23 ± 1°C and 65 ± 5% RH). Bees were maintained without food to ensure they were motivated to feed, but they were given access to water in a small dish with cotton plug. On the day of the experiment, each bee was provided with corresponding treatment solution ad libitum using a 1.7 ml Eppendorf tube (two tubes per box) with a hole that provided easy access to the feeding solution by the bees. The tube was positioned at a downward angle to reduce the likelihood that a bubble would form over the feeding hole. A set of five treatment boxes with only sucrose solution and without a bee were used to control for evaporation. The experiment was conducted in a climatic chamber (16 : 8 h L : D, 23 ± 1°C and 65 ± 5% RH) with daily observations of survival and food consumption for 6 days. Eppendorf tubes were refilled daily with their respective fresh treatment solutions. A total of 90 bees (15 bees per treatment) were used for the experiment.

### 2.5. Laboratory-based macronutrient assay—paired diets

To test our hypothesis that *M. rotundata* bees’ intake targets lie between the 10 : 1 and 1 : 1 P : L ratios (see §3.2), we measured the nutrient consumption of bees using paired diets that spanned P : L ratios that ranging between 25 : 1 and 1 : 1. Following the methodology described in the above single-diet assay, we caged individual bees and offered them one of three paired P : L combinations: (i) 25 : 1 and 5 : 1, (ii) 10 : 1 and 5 : 1 and (iii) 1 : 1 and 5 : 1. Over 6 days, we monitored bee survival daily and measured consumption of each diet and nutrients on alternate days while adjusting for evaporation rates (figure 1C). A total of 60 bees were used for the assay (*n* = 20 bees per treatment). Consumption of food and nutritional contents were analysed for bees that survived for the entire 6 days of the experiment.

### 2.6. Statistical analysis

All statistical tests and plotting were performed in R v. 3.5.0 (R Foundation for Statistical Computing, Vienna, Austria).

For the semi-field trials using *Capsella* plants, non-parametric Spearman correlations were used to assess the relationship between nutritional metrics (protein concentration, lipid concentration and P : L) and bee visitation (CVR and IVD) on scaled data.

For the laboratory studies, to determine whether leafcutting bees fed randomly among diet sources or if any treatment diets caused differential feeding behaviour, we analysed differences in daily consumption of diet sources among treatments by two-way ANOVA and *post hoc* Tukey honest significant difference (HSD) pairwise comparisons with treatment, day and the interaction of treatment and day as independent variables, and the initial bee body mass as a covariate. To analyse differences in daily consumption of nutrients among treatments, we used non-parametric permutation multivariate analysis of covariance (PMANCOVA) with *post hoc* Tukey HSD pairwise comparisons with nutrient (carbohydrate, protein or lipid) as the dependent variable and body mass as a covariate. For paired-diet assay, to determine whether there were any differences in food consumption based on P : L of the diet source, an additional interaction between the treatment and diet source was included in the model. If the total consumption of nutrients was consistent across treatments (in paired diets), it would indicate that the *M. rotundata* leafcutting bees were effectively regulating their individual nutrient intake to meet their target nutritional needs. We determined P : L ratios consumed by bees using the average cumulative consumption of each treatment.

To evaluate the impacts of specific macronutrient ratios on bee survival, we established Kaplan–Meier survival curves for each of six single diets and three paired-diet treatments with log-rank comparisons between treatments.

## 3. Results

### 3.1. Bees preferentially visit *Capsella* plant lines based on their pollen nutritional content

Foraging preferences of *M. rotundata* leafcutting bees were evaluated for 16 RILs and two parental lines, *Cg* and *Cr*, in two semi-field cages. A total of 2326 single visits were recorded by 171 active female bees through 16 outdoor foraging cage trials.

For each plant line, both theCVR and IVD were recorded. Female *M. rotundata* preferentially visited the pollinator-dependent *C. grandiflora* parental line over selfing *C. rubella* parental line. There was also a significant variation in their visitation preferences among the RILs (electronic supplementary material, figure S2). RIL 49 and RIL 90 exhibited the highest attractiveness (comparable to *Cg*), whereas RIL 148 was the least attractive to *M. rotundata* in terms of CVR (see electronic supplementary material, figure S3).

**Figure 2.**
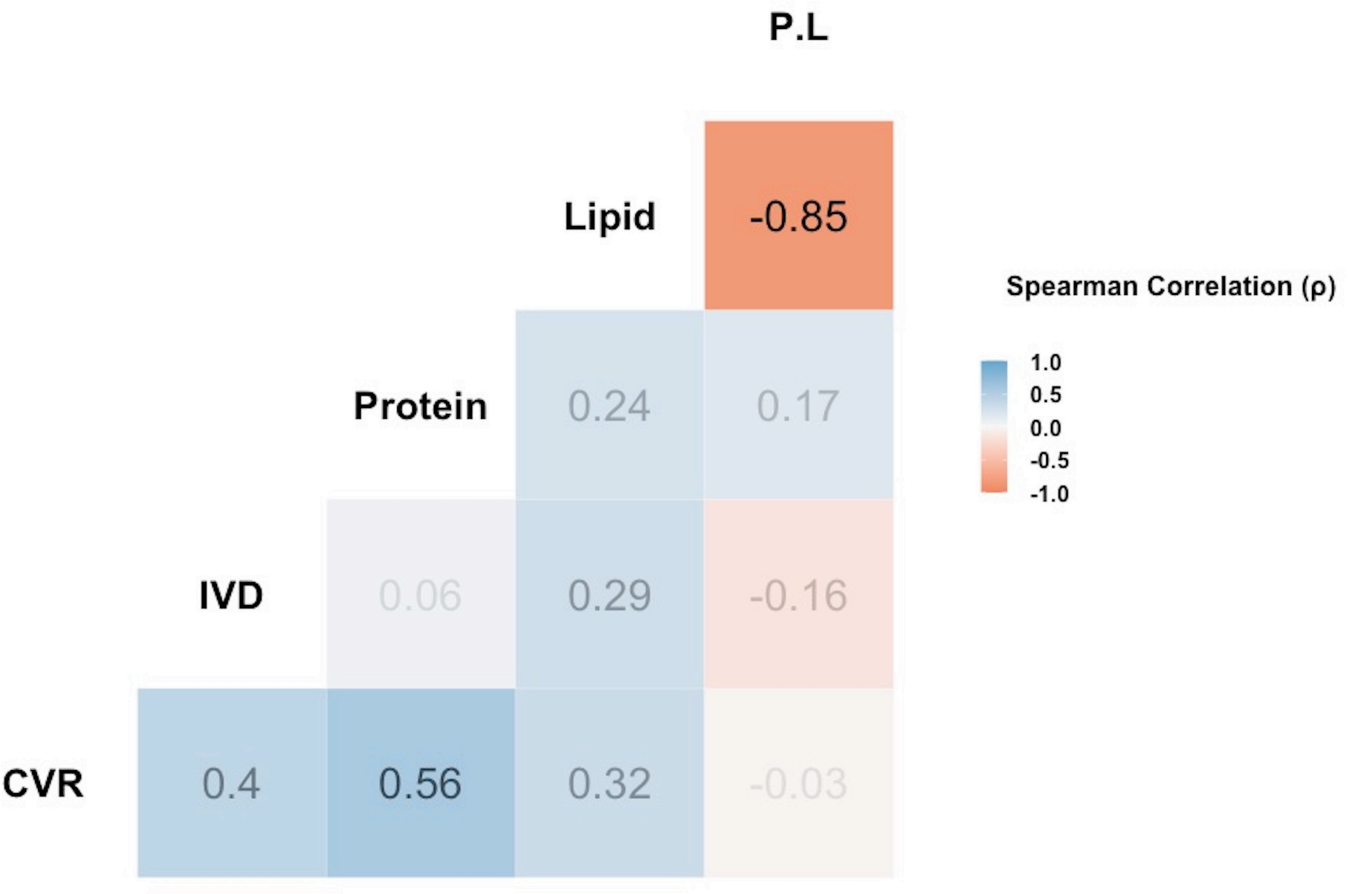
Spearman correlation plot among nutritional factors and visitation rates for the leafcutting bee species, *Megachile rotundata*, foraging on the recombinant *Capsella* inbred plant lines. These lines were produced by crossing an obligate outcrosser and insect-pollinated plant (*Cg*) with a self-fertilizing line (*Cr*). For each foraging trial, 20 female *M. rotundata* were released into the field cage containing 18 *Capsella* plant lines (16 recombinant lines and two parents, *Cg* and *Cr*), and bee foraging was observed for 2 hours. A total of 16 trials were conducted in two cages. Protein and lipid concentrations in pollen were estimated for each *Capsella* plant line (see electronic supplementary material, table S1 and [24]). Analysis was conducted on the average values for each variable factor. Values indicate pairwise Spearman correlation coefficients. CVR, Cumulative Visitation Rate; IVD, Individual Visit Duration. A significant positive correlation was observed between CVR and protein concentration in pollen.

Figure 2 shows the correlation between factors (i.e. CVR, IVD, protein concentration, lipid concentration and P : L). The CVR among *Capsella* plant lines was positively correlated with the protein concentration (Spearman: rho = 0.56, *p* = 0.01; figure 2) and was not correlated with the lipid concentration in pollen (rho = 0.32, *p* = 0.11). Consistent with our previous studies conducted in indoor arenas using a single cut inflorescence for each *Capsella* plant line [24], these outdoor cage experiments with whole plants again demonstrated that the leafcutting bees were generally attracted to RILs with higher P : L ratios (with RIL 90 and RIL 49 that have 8.6 : 1 and 5.6 : 1 P : L respectively, being the most attractive lines) (electronic supplementary material, figure S3), reinforcing the role of protein in enhancing collective foraging activity. However, IVD did not significantly correlate with protein concentration, implying that while bees visited protein-rich resources more frequently, their per-visit engagement remained consistent. Nonetheless, a non-significant but slightly positive correlation was observed between IVD and lipid concentration (rho = 0.29, *p* = 0.24), suggesting a potential trend towards longer visits on lipid-rich flowers, possibly reflecting increased handling time or pollen collection effort for each visit.

### 3.2. Macronutrient ratios—single-diet assay

**Figure 3.**
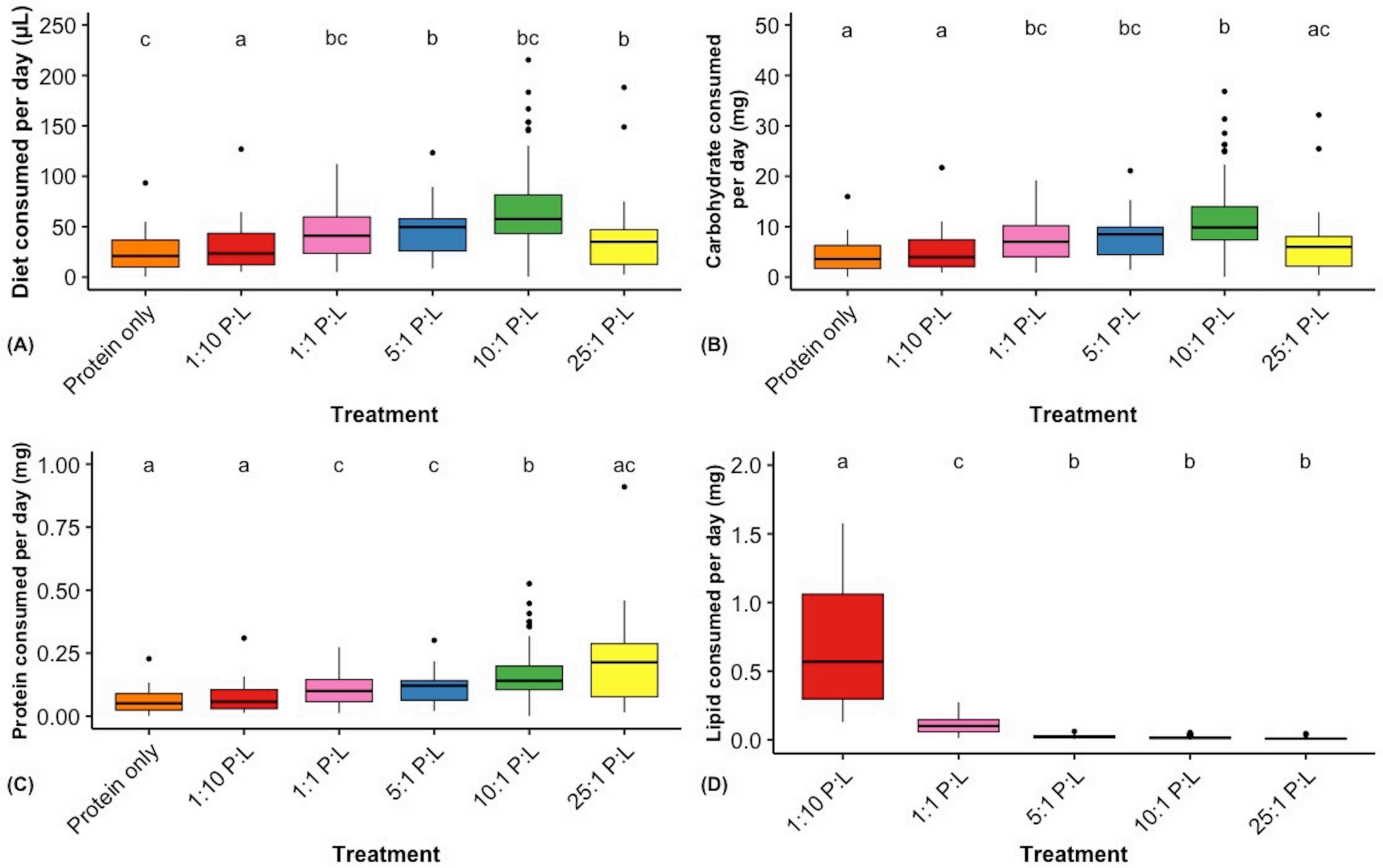
Box plots for the daily consumption of (A) total diet and individual macronutrient components, (B) carbohydrates, (C) protein and (D) lipids, for female alfalfa leafcutting bees, *Megachile rotundata*. Pairwise comparisons were made using Tukey HSD *post hoc* analysis using log-transformed data for each variable. The sample sizes for each treatment group were as follows: Protein only (*n* = 58), 1 : 10 P : L (*n* = 35), 1 : 1 P : L (*n* = 57), 5 : 1 P : L (*n* = 67), 10 : 1 P : L (*n* = 74) and 25 : 1 P : L (*n* = 55).

We provided *M. rotundata* females with ad libitum access to one of the six P : L diets; protein only, 25 : 1, 10 : 1, 5 : 1, 1 : 1, 1 : 10 P : L, for 6 days. The total daily food consumption significantly differed among the treatments (F_5,309_ = 16.06, *p* < 0.0001), regardless of day (*p* = 0.12) and bee body mass (*p* = 0.46). However, interaction between diet treatments and day of observation was significant (treatment*day: F_25,309_ = 2.51, *p* = 0.0001) (see electronic supplementary material, figure S4). Similar to the semi-field experiment (which showed highest preference for RIL with 8.6 : 1 P : L), this experiment found the highest consumption on the 10 : 1 diet, followed by 5 : 1 and 1 : 1 P : L treatments (figure 3A).

The daily consumption of individual macronutrients (carbohydrates, proteins and lipids) in *M. rotundata* exhibited significant variations across treatments (PMANCOVA: *p* < 0.0001). Notably, bees fed with 10 : 1 P : L diet consumed significantly greater amount of carbohydrates (F_5,309_ = 18.0; *p* < 0.0001; figure 3B) and proteins (F_5,309_ = 18.0; *p* < 0.0001; figure 3C) compared with other treatments. Bees on the highest lipid diet (1 : 10 P : L) consumed significantly more lipid (mean ± s.d.: 0.72 ± 0.61 mg) and much less protein (0.07 ± 0.06) than the other treatments (F_5,309_ = 30.3; *p* < 0.0001; figure 3D), suggesting that these bees may have ceased feeding once their lipid intake targets were met or exceeded, thereby failing to achieve their protein requirements.

In addition, the consumption of each macronutrient by *M. rotundata* bees varied across days with treatment interaction (*p* < 0.001; see electronic supplementary material, figure S4). Only bees fed with 10 : 1 P : L diet showed increasing consumption of diet, protein and carbohydrates over the 6 days of the experiment, while the bees fed the other diets showed declining consumption. This suggests that the other diets allowed the bees to achieve their preferred protein concentration more rapidly, or the other diets contained too high levels of lipids.

In addition, bees with greater initial body mass consumed less lipids (but not carbohydrates or proteins) over days of feeding (*p* = 0.03), indicating that heavier bees tend to use their lipid reserves rather than accumulating additional ones.

**Figure 4.**
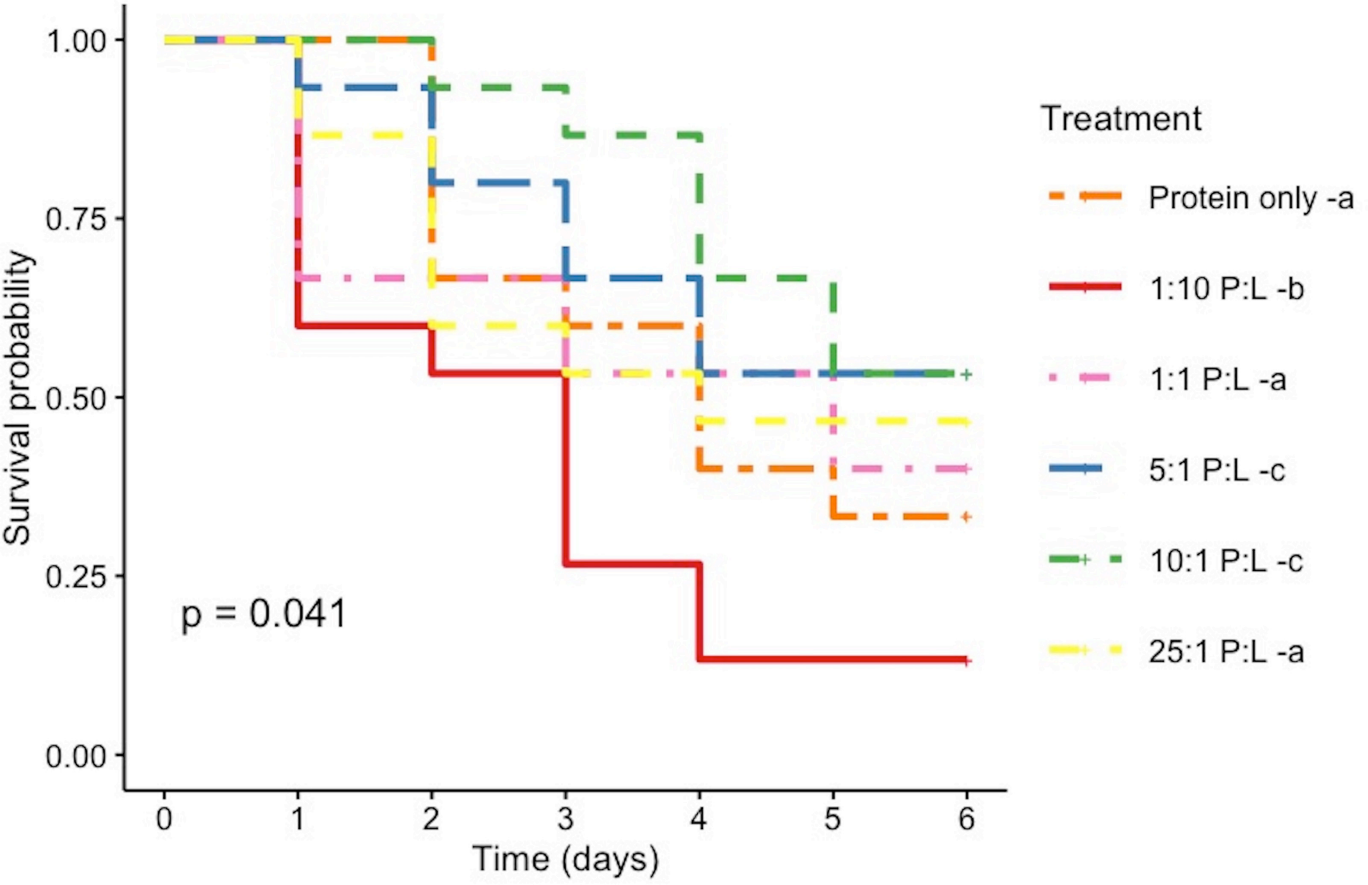
Kaplan–Meier survival curves for the single-diet treatments using artificial feeders for female alfalfa leafcutting bees, *Megachile rotundata*. The plot illustrates the survival probability over time (days) in each treatment. Survival distributions between groups were compared using the log-rank test. Different lowercase letters after treatments (in legend) indicate pairwise significant differences.

Furthermore, bees that fed with 10 : 1 and 5 : 1 P : L showed significantly higher survival rates compared with other treatments (log-rank *p* = 0.04), while over 50% of 1 : 10 P : L fed bees died within 3 days of treatment (figure 4).

### 3.3. Macronutrient ratios—paired-diet assay

Over a period of 6 days, we fed *M. rotundata* females ad libitum with a 5 : 1 P : L diet alongside a complementary diet (25 : 1, 10 : 1, or 1 : 1 P : L). Each combination of the 5 : 1 P : L and treatment P : L established a protein and lipid nutrient space encompassing the hypothesized P : L intake target. Similar survival rates were observed across the treatments (log-rank *p* = 0.11), with slightly decreasing survival rates for the 1 : 1 and 5 : 1 P : L paired-diet treatment (see electronic supplementary material, figure S5).

The *M. rotundata* bees exhibited significant differences in total food consumption across the treatments (F_2,226_ = 3.07, *p* < 0.05) and with the interaction between treatment and diet sources (F_3,226_ = 35.9, *p* < 0.0001). Importantly, daily consumption patterns revealed significant differences between the treatment diets (25 : 1, 10 : 1 and 1 : 1) and the 5 : 1 diet, suggesting that the bees were making selective feeding choices rather than consuming the diets at random (electronic supplementary material, figure S6). Such selective behaviour implies a targeted approach to nutrient intake, highlighting the bees’ ability to discern and prefer certain dietary compositions based on their nutritional needs.

**Figure 5.**
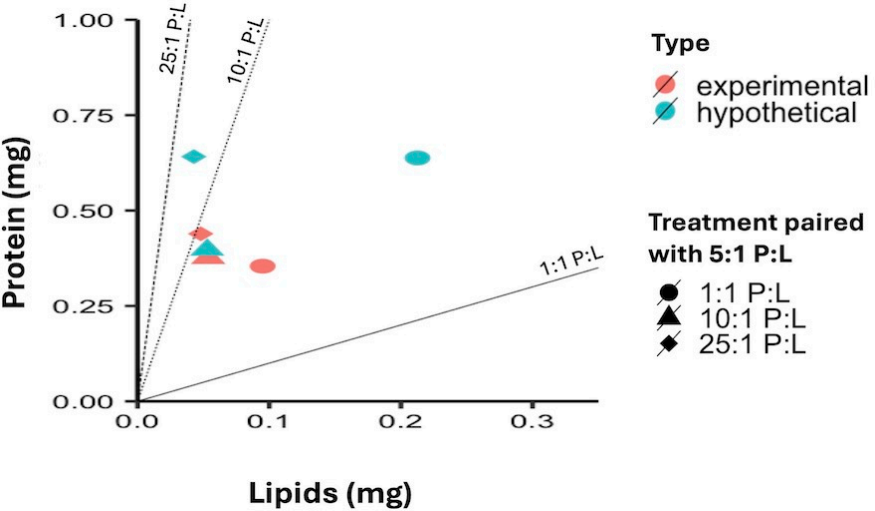
Comparison of average P : L concentration ratios in the paired diets consumed by female alfalfa leafcutting bees, *Megachile rotundata*, using artificial feeders. Bees were provided with two feeders: one with a constant 5 : 1 P : L ratio in all treatments, while the other feeder had a 1 : 1, 10 : 1 or 25 : 1 P : L ratio depending on the treatments. Hypothetical concentrations for equal consumption of both diets (green) are contrasted with the experimentally observed P : L concentrations (red) for each treatment. The plot illustrates the differences in the P : L intake based on the bees’ actual consumption behaviour relative to the predicted values.

Among the macronutrient components, the surviving bees exhibited consistent consumption of carbohydrates and proteins (*p* > 0.1), while lipid consumption varied significantly across the treatment groups (F_2,227_ = 23.3, *p* < 0.0001). Nonetheless, total nutrient intake remained consistent across the different treatment groups (*p* > 0.1, electronic supplementary material, figure S7), suggesting that the *M. rotundata* bees regulated their P : L intake to within our hypothesized range (25 : 1 to 1 : 1 P : L). To calculate the hypothetical P : L intake for each pair diet treatment, assuming no preferences or selective feeding, we assumed an average consumption of 70.5 µl per day for the diet, with equal intake in both diets of a paired treatment. Figure 5 shows the average P : L consumption among treatments, visually comparing the experimental P : L ratios with the hypothetical values. The experimental P : L ratios were much closer to one another among the treatments (average ± s.e.: 6.6 : 1 ± 1.6 : 1 P : L), aligning with preferences observed in the semi-field and single-diet assays (between 10 : 1 and 5 : 1 P : L), indicating a more consistent nutrient profile across the diets. This regulation of nutrient intake, regardless of treatments, suggests adaptive feeding behaviour to achieve a nutritional intake target.

## 4. Discussion

In our previous laboratory-based foraging study, using *Capsella* recombinant plants, we demonstrated that *M. rotundata* leafcutting bees preferentially visit flowers based on the protein and lipid concentrations of the pollen [24]. In the current study, we demonstrate that, when *M. rotundata* forages on whole plants, they preferentially visit *Capsella* RILs with relatively higher pollen protein concentrations (CVR), though, once they select a flower to forage on, they spent the same amount of time on all flowers regardless of pollen nutritional content (IVD). When considered CVR and IVD data together, our results suggest that protein content drives overall foraging activity and resource selection, rather than modulating the micro-behaviour of individual bees during each visit. This is consistent with findings in bumblebees and honeybees, where pollen protein concentration has been shown to influence recruitment and foraging allocation without necessarily affecting per-visit handling time [11,29]. Our experiments using artificial diets with varying P : L ratios revealed that female *M. rotundata* exhibit altered feeding behaviour impacting survival (single-diet assay) and regulate their nutrient intake to maintain an average of 6.6 : 1 P : L (±1.6 s.e.) (paired-diet assay). This preferred ratio is consistent with studies examining the P : L ratios of pollen collected for brood provisions of nesting *M. rotundata* bees [30]. Notably, bees’ survival was highest when fed diets with 5 : 1 and 10 : 1 P : L, and mortality increased with higher lipid diets (figure 5). Altogether, these findings provide compelling evidence that *M. rotundata* mated female bees actively regulate their intake of macronutrients, adjusting the P : L ratio in diet to optimize survival (and possibly health [31]).

Recent studies have suggested that different bee species exhibit selective foraging behaviour to obtain pollen with specific P : L ratios [3,4,6,28,32], reflecting their individual nutritional needs and preferences. However, foraging preferences can be influenced by the bees’ foraging availability—for example, bees may forage on the plant that is most abundant—and thus detailed studies evaluating nutritional preferences in field, semi-field and laboratory conditions are needed to determine if an individual species is indeed foraging according to the NGF [33]. In our semi-field study using whole flowering plants, *M. rotundata* bees showed preference for flowers containing high concentrations of protein in their pollen (see electronic supplementary material, table S1 for macronutrient concentrations, [24]). In our previous laboratory-based studies using isolated inflorescences from these plants, *M. rotundata* bees showed a preference for flowers with pollen with high concentrations of protein [24]. In our laboratory-based study using synthetic liquid diets, *M. rotundata* showed a clear selective preference for diets with a 1 : 1 to 10 : 1 P : L ratios. However, only diets in the 10 : 1 and 5 : 1 P : L supported both high consumption and survival, indicating that these represent the functional nutritional optimum, while 1 : 1 P : L may be acceptable but not sufficient for long-term performance. When allowed to self-select a diet, these bees preferentially fed to achieve a mean diet of 6.6 : 1 P : L (figure 5), closely matching the average P : L ratio of 7 : 1 found in natural pollen provisions collected by wild *M. rotundata* female bees for forming their brood provisions in their nests [30]. This alignment suggests that adult nutritional regulation may be finely tuned to the nutritional needs of their progeny. Given that solitary bee larvae consume only what their mothers provision, the adult’s dietary preferences probably reflect evolutionary optimization for offspring development [11]. Thus, adult survival responses under defined P : L diets can serve as a meaningful proxy for understanding larval nutritional requirements in solitary bees. Overall, these studies demonstrate that *M. rotundata* bees selectively forage to collect pollen with a near 6.6 : 1 ratio under a variety of environmental conditions. Nevertheless, direct studies measuring larval growth, survival and fitness outcomes across controlled P : L diets are needed to confirm whether maternal nutrient regulation translates into optimal larval performance. Shik *et al.* [34] observed a comparable pattern in fungus-farming ants, which regulated carbohydrate and protein inputs to optimize their fungal cultivar growth, avoiding excesses that trigger harmful resource allocation. Future research integrating both maternal behaviour and offspring developmental outcomes will be essential to fully understand nutritional regulation across generations in solitary bees and other insects.

In paired-diet assay, we specifically observed that while lipid consumption varied significantly across treatments, protein and carbohydrate intake remained relatively similar, indicating that bees modulated their lipid intake to maintain a consistent nutritional profile. The deviation from hypothetical values (assuming no regulation) and clustering around intermediate P : L ratios (averaging at 6.6 : 1) further support the hypothesis that bees selectively forage to meet a macronutrient target, a hallmark of regulatory behaviour within the nutritional geometric framework [1]. These findings are consistent with earlier work on bumblebees [5] and extend the evidence for nutrient regulation to solitary bees, which comprise most bee species but remain under-represented in nutritional ecology studies.

The foraging and feeding preferences of a small number of other bee species have been investigated with detailed laboratory and field studies. *Bombus terrestris* and *B. impatiens* bumblebees regulated their diet intake to achieve an average 14 : 1 and 12 : 1 P : L, respectively, when evaluated with synthetic liquid diets in the laboratory, but in semi-field cages with selected flowering plants and in field studies [15], bees collected pollen with a 4 : 1 P : L ratio, suggesting they may be limited in their ability to access high P : L pollen in the field conditions [5]. When examining the nutritional content of pollen collected by foraging bees either returning to the nest or on flowers in the field, different bee species have been found to preferentially forage on or collect pollen with species-specific averages ranging from 0.4 : 1 to 12.5 : 1 P : L [3,6,35], but detailed studies using controlled foraging conditions in the laboratory and in semi-field conditions are needed to determine if these preferences are due to species-specific preferences or forage availability in the environment. While pollen specialists (oligolectic) are probably nutritional specialists due to their restricted host range, it remains unclear whether pollen generalist (polylectic) species, despite collecting pollen from a wide variety of plants, exhibit consistent nutrient regulation and function as nutritional specialists, or whether they are true nutritional generalists. Moreover, this selective foraging behaviour can be influenced by factors such as the bee’s physiology and life-history traits [17,36–38] and thus bees under different conditions may show different nutritional preferences.

In social bees, such as honeybees and bumblebees, colony-level modulation of P : L ratios may allow for more flexibility in managing nutritional intake, with worker bees foraging collectively and providing resources to larvae and the queen [38]. In this context, P : L ratios need to be adjusted based on colony demands, with some degree of compensation or redistribution of resources across brood stages and individuals [39,40]. In contrast, solitary bees, like *M. rotundata*, lack this social structure and, therefore, cannot adjust the nutritional profile of resources over time. As a result, their foraging and provisioning strategies may be more rigid, potentially making them more sensitive to changes in P : L ratios, particularly when protein or lipid levels deviate from the optimal range for survival or larval development, thus may exhibit stronger dependencies on precise nutritional balances. Our results for *M. rotundata* are in line with this notion, as female survival was influenced by P : L ratios, emphasizing the more precise and less flexible nutritional needs of solitary bees.

The optimal diet can vary depending on the metric that is being assessed—for example, different diets may support development, growth, reproduction and survival [10]. Here, we evaluated survival of adult female *M. rotundata* and found the highest survival rates in bees fed diets with P : L ratios of 5 : 1 and 10 : 1. In *B. terrestris* and *B. impatiens* adult female bumblebees, the best survival was at 10 : 1 P : L for both species. For both bumblebees [5] and *M. rotundata* (our study), although the exact mechanisms remain unclear, the observed increase in mortality risk associated with lipid content exceeding a 5 : 1 P : L ratio highlights the potential negative effects of excessive lipid intake on bee/colony health [41]. Bees might prioritize nutrient intake to support egg production, development and/or health [19,42,43], with an increased demand for lipids and proteins required for reproduction [38]. If we consider immune function, nutrient intake could be skewed towards specific amino acids or lipids that support immune responses [11,44–47], to support the synthesis of antimicrobial peptides or fat stores that help cope with pathogen challenges [48]. These variations underscore the complexity of nutrient regulation and the need to consider different physiological demands when assessing optimal nutrient intake in bees.

How bees are assessing the nutrient content of their diets remains to be determined. In our study, *M. rotundata* bees in the high-protein (25 : 1 P : L) or high-lipid (1 : 10 P : L) treatments initially consumed high concentrations of either nutrient, which probably contributed to their rapid mortality. It appears that the surviving individuals were able to ingest enough to meet their nutritional requirements, recognize the toxicity of the diet and subsequently reduce or cease feeding. In contrast, the bees that did not survive probably failed to regulate their intake appropriately. The underlying factors contributing to this individual variation in behaviour remain to be determined.

Some insect species aim to select and consume diets in a way that brings their nutrient intake as close as possible to an optimal balance, minimizing the ‘distance’ from an ideal P : L ratio (or other nutritional targets) for their individual or colony needs. For instance, the regulation of nutrient intake was experimentally investigated in *Locusta migratoria* (L.) nymphs by offering them 25 artificial foods, each with varying levels of protein and digestible carbohydrate [49], recorded intake and growth, and constructed two-dimensional plots for the intake and nutritional targets. Analyses revealed that locusts regulated both protein and carbohydrate intake with equal efficiency, consistently choosing foods that brought their nutrient intake closest to the optimal target, following a principle of ‘closest distance optimization’. This suggests that locusts employ a functional strategy to precisely regulate their nutrient intake, aiming to maintain a balanced diet by minimizing the deviation from their nutritional goals. In another study comparing the nutrient balancing strategies of two grasshopper species, *Locusta migratoria* (a grass-specialist) and *Schistocerca gregaria* (a desert-generalist), although both species maintained a balanced intake of protein and carbohydrate when given complementary foods, the generalist selected more protein than the specialist and exhibited greater flexibility in response to nutrient imbalances [50]. The desert locust *S. gregaria* species also showed better survival and development when consuming higher-protein diets [50], suggesting that generalist species have greater behavioural and physiological adaptability to dietary imbalances than specialists.

Our findings contribute to a growing understanding of how nutritional regulation shapes bee foraging behaviour, with important implications for pollinator ecology and conservation [11,50,51]. By demonstrating that solitary bees respond to variation in plant macronutrient profiles, this study supports the value of incorporating nutritional traits, such as P : L ratios, into pollinator-friendly planting and habitat restoration strategies [11,52]. This is especially relevant for solitary bees with specialized plant associations, whose foraging options may be restricted to a narrow range of floral resources [33,53,54]. If those plants provide nutritionally imbalanced pollen, the bees may face developmental or reproductive constraints. Consequently, our results highlight the need to consider nutritional traits when designing conservation strategies and habitat restorations, especially for nutritional specialist species or bees in fragmented landscapes. This is particularly relevant in agricultural settings where floral diversity is often reduced and may be dominated by pollen sources with imbalanced or suboptimal nutrient content [55]. Ensuring the availability of nutritionally diverse and balanced floral resources could help support bee health, improve reproductive success and buffer populations against environmental stressors [53,56]. This nutritionally informed approach is essential for supporting both generalist and specialist pollinators across diverse ecosystems.

## 5. Conclusions

Integration of laboratory, semi-field and field studies has demonstrated that alfalfa leafcutting bees (*M. rotundata*) selectively forage across diverse flowering plant species to collect and/or feed on diets with a specific macronutrient ratio, and preferred diets are associated with improved survival of adult female bees. However, one other bee species in the Megachilidae family, namely the horn-faced mason bee (*O. cornifrons*) shows a broader dietary preference and forages according to availability [24]. These studies lay the groundwork for comparative studies examining the tendency for nutritional specialization versus generalization across bee species, how developmental and physiological conditions influence foraging and dietary preferences, and the molecular, physiological and neurobiological processes underlying these foraging and feeding preferences.

## Data Availability

Data for these studies are available through the Pennsylvania State University’s ScholarSphere data repository [57]. Supplementary material is available online [58].
